# Pilot implementation outcomes of a community-based tele- practice model for identification and rehabilitation of children with hearing loss within a public-health system of a Rural District in Southern India

**DOI:** 10.1371/journal.pone.0319109

**Published:** 2025-03-19

**Authors:** Karishmaa Satheesh, Vidya Ramkumar, Deepashree Joshi, Bommi E.

**Affiliations:** 1 Department of Audiology, Sri Ramachandra Faculty of Audiology and Speech Language Pathology, Sri Ramachandra Institute of Higher Education and Research (DU), Porur, Chennai, Tamil Nadu, India; 2 Department of Audiology, SRESHT Lab, Sri Ramachandra Faculty of Audiology and Speech Language Pathology, Sri Ramachandra Institute of Higher Education and Research (DU), Porur, Chennai, Tamil Nadu, India; 3 District Differently Abled Welfare Officer, Perambalur, Perambalur District, Tamil Nadu, India; All India Institute of Speech and Hearing, INDIA

## Abstract

**Background:**

The current study is an effort to evaluate pilot outcomes of a comprehensive tele-practice model for identification and rehabilitation of hearing loss among children below six years of age, which can then inform suitable adaptations prior to the implementation. The outcomes of tele-facilitator training, limited-efficacy measures, and caregiver acceptability and satisfaction with tele-practice were analyzed.

**Method:**

Two special educators were trained as tele-facilitators for tele-diagnostic testing. Screening was done using the validated SRESHT screener in the Perambalur district by trained nurses in the Upgraded Primary Healthcare Centers at all four blocks until at least five children with ‘refer’ results were obtained. For tele-rehabilitation, we enrolled five children with hearing loss who used amplification devices and attended the District Early Intervention Centre. To measure the limited- efficacy, the outcomes of pre-pilot and post-pilot were compared. The caregiver acceptability and satisfaction with tele-practice were also assessed.

**Results:**

In all 12 children (age range of 5 months to 6 years; 6 male and 6 female) with ‘refer’ results in hearing screening underwent tele- diagnostic testing. Tele-rehabilitation outcomes were studied on 5 children with hearing loss (age range of 4 years to 6 years; 3 male and 2 female) who had already used amplification devices. When comparing the outcomes of limited-efficacy with the existing data, it was found that the model identified older children with hearing loss. The time lapse between screening/hearing loss suspicion and diagnosis reduced from a median of 216 days to eight days. For tele-rehabilitation, the number of sessions attended in a span of three months increased from a median of zero to three sessions. Based on the satisfaction questionnaire, most people had a positive experience and found the travel time to the testing site convenient and affordable. Few parents reported encountering difficulties as a result of inadequate ventilation and internet connectivity within the mobile van where tele-diagnostic testing was conducted.

**Conclusion:**

The pilot outcomes suggest that a block-level service enabled using tele-practice to overcome professional shortages was beneficial in reducing time gap between screening/suspicion and diagnosis and also enhanced attendance for rehabilitation. The pilot outcomes provided insights on adaptations related to screening sites, test infrastructure, and internet optimization that may be required before implementation.

## Introduction

Hearing loss in childhood can significantly impact the development of spoken language, as well as societal inclusion, access to information and knowledge, and eventually desirable academic and vocational academic outcomes. To reduce the impact of hearing loss, it is pertinent to detect congenital hearing loss before 3- 6 months of age [1] through newborn hearing screening programs and also introduce school screening programs to mitigate the consequences of unidentified late onset and other preventable hearing losses.

In 2013, the Government of India launched a national program to screen and identify any defects, diseases, deficiencies, and disabilities among children from zero to 18 years of age in order to encourage early identification of disabilities. A health team, at the village level, led these screening efforts, referring individuals who fail the screening to the Early Diagnostic Centre (EDC) at the district General Hospital (GH) for diagnosis. Further, post confirmation of disability, the individual is directed to the District Differently Abled Welfare Office (DDAWO) for welfare benefits, aids and appliances, and early intervention. In 2015, the ‘Mobile Therapy Unit’ was implemented through the State Commissionerate for the Differently Abled to improve consistency of rehabilitation services, increase last-mile coverage, and overcome the challenge of patient’s access to services. This service is provided with existing professionals available through District Differently Abled Welfare Office or local non-governmental organizations (NGOs). However, this initiative is also challenged by higher demand on professional’s time who are engaged in both clinical services and administrative work. A recent situational analysis confirmed this mismatch of demand versus need [2].

Tele-practice/ tele-health has been explored as one of the options to overcome the challenges of demand versus capacity and access barriers. There is a growing body of evidence on the use of telehealth in hearing health care, including screening [3], diagnostic assessment [4], hearing aid fitting [5] and rehabilitation [6]. With more than 3.74 billion smartphones and 4.92 billion mobile phones worldwide, mHealth applications are showing potential to increase access to low- and middle-income countries (LMICs) [10]. Several studies from Africa, the United States, and India have shown promising outcomes in reaching the rural community through tele-practice for individuals with hearing and speech-language disorders [7–9,11–13]. Despite these, there have been only a few attempts to investigate a comprehensive diagnostic audiology solution [7,14,15]. Even these, however, have focused only on diagnostic testing and did not include rehabilitation. Furthermore, only a few studies in a high-income country examined the outcomes of implementing tele-audiology in a health care system [7]. There are no such reports from the low-middle income country context.

Therefore, we developed a comprehensive tele-practice model to identify and rehabilitate children with hearing loss under six years of age in rural communities, and integrated it into public-sector health services in a rural district in Southern India. The proposed intervention is complex as it involves multiple steps (as stated above), multiple departments (District Differently Abled Welfare Office, Early Intervention Centre, Non-Government Organizations), and many stakeholders at various stages of implementation (Audiologist and Speech Language pathologist, child with disability, caregivers, parents, nurses, special educators, government officers, non-professional staff). The intervention necessitates that stakeholders adopt technology to a greater extent than is currently used, thereby altering their work process. We identified and mapped implementation characteristics to the Consolidated Framework for Implementation Research (CFIR) [16]. The model was designed for implementation in four blocks of a rural district (Perambalur Block, Veppanthattai Block, Alathur Block, and Veppur/Kunnam Block). Prior to the model’s finalization, several stakeholders from the State Commissionerate for the Welfare of Differently Abled, Departments of Health, Social Justice and Welfare, and Education, as well as parents of children with and without disabilities, were involved to ensure model optimization and convergence of efforts toward early identification and intervention [2].

**Fig 1:**
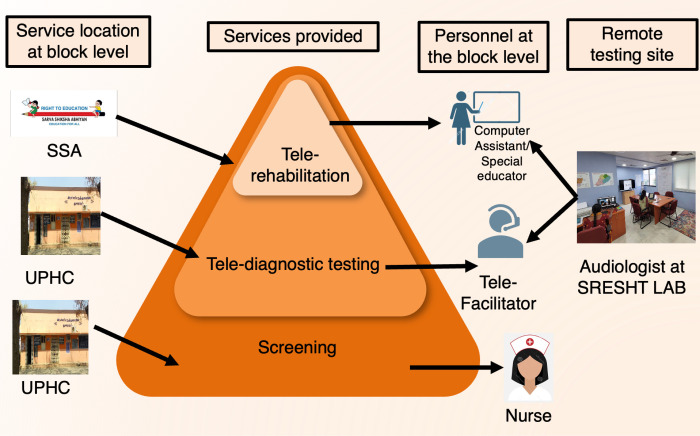
Comprehensive Tele-practice model.

In this model, nurses in Upgraded Primary Health Centers were to be trained to screen for hearing impairment using a validated tablet-based screening device (see https://sresht.godaddysites.com/resources) in order to triage disabling hearing losses (>=60 dB HL) as defined in the RPWD Act 2019. For individuals who ‘refer’ the hearing screening, tele-diagnostic testing would be delivered remotely from the university’s SRESHT lab to children in a mobile-van with an audiology test facility at each of the four block’s Upgraded Primary Healthcare Centers. Staff from NGOs or government Early Intervention Centres served as tele-facilitators. Finally, individuals identified were to receive tele-rehabilitation services at the Block Resource Centers of the ‘Education for All’(Sarva Shiksha Abhiyan) scheme of the government of India (SSA-Block Resource Center), with the assistance of computer assistants/SSA teachers and under the supervision of a remote audiologist/speech language pathologist/auditory verbal therapists (Fig 1).

The aim of the current study was to evaluate the pilot outcomes of this comprehensive tele-practice model. The objectives were: (i) to evaluate the outcomes of training tele-facilitators to assist in remote diagnostic hearing assessment and rehabilitation services; (ii) to evaluate the limited -efficacy outcomes of the pilot. Limited-efficacy measure is defined as an analysis that uses a convenience sample, intermediate rather than final results, shorter follow-up periods, or has limited statistical power [17]. Additionally, it involves evaluating the patient’s acceptance and satisfaction with the tele-diagnostics and telerehabilitation components of the comprehensive tele-practice model.

## Method

### Ethics approval and consent to participate

This study received ethical clearance (IEC-NI22/DEC/85/124) from the Institutional Ethics Committee (IEC) in Sri Ramachandra Institute of Higher Education and Research (Deemed to be University). Informed written consent was obtained from the respective parents of children who participated, prior to the study.

### Study site

The local government selected the pilot implementation site (Perambalur district) for this project. This district has an area of 1,757 sq. km. The population per block includes 1,17,534 in Veppur, 5,692 in Perambalur, 1,48,743 in Veppanthattai, and 1,57,947 in 7 Alathur blocks, respectively. We implemented the pilot for three months, from 1st April 2023 to 1st July 2023.

***Study design:*** Quasi-experimental within-site design

### Study subjects

#### Sample for tele-diagnostic evaluation.

Hearing screening was done using the validated SRESHT screener for children below 6 years in the communities of Perambalur district by trained nurses working in Upgraded Primary Healthcare Centers at all four blocks of the district until at least five children with ‘refer’ results were obtained between 1st April to 1st July 2023. These children were then referred for tele-diagnostic evaluation.

#### Sample for tele-rehabilitation.

In general, all children who undergo cochlear implantation/ hearing aid fitting are recommended for in-person auditory training and special education at the district Early Intervention Centre. We used the convenience sampling method to include five children with hearing loss who were enrolled in the District Early Intervention Centre and were using amplification devices (four children using unilateral CI and one child using bilateral hearing aids) for tele-rehabilitation.

### Procedure

The procedure is explained in a sequence of four steps

#### Step 1: Training and assessment of the tele-facilitator’s knowledge and skill.

The District Differently Abled Welfare Office deputed a special educator and a trainee special educator for hearing impairment (HI) to train them as tele-facilitators to assist with tele-diagnostics and tele-rehabilitation. Three audiologists conducted the training over two days, for five hours per day. The training primarily covered aspects of internet connection, the use of the TeamViewer application (version 15.40.8) to connect remotely to the audiologist, the purpose of the test, the setup of equipment in the mobile van, instructions to the parent on the test procedure, patient preparation for testing, equipment connections, and the placement of transducer, probe, and electrodes on the patient.

During the training period, we conducted a hands-on training session to acquaint them with the software, equipment connections, and placement of transducer/probe/electrode. At the end of the training session, a procedure manual (see S1 file) along with a video demonstration developed in English and local language (Tamil) (available at https://www.youtube.com/watch?v=xEKYm4sKnWg)

A questionnaire (see S2 file) was used to assess the tele-facilitator’s knowledge and an Objective Structured Clinical Examination (OSCE) (see S3 file) and a labeling form with images of the equipment were used to assess the skill (see S4 file). We developed the questionnaire for knowledge assessment using Bloom’s taxonomy [18] and included 10 close-ended questions. Five of the questions were about technical factors such as internet connection, audio-video call, and troubleshooting, while the other five were about audiological test processes. The OSCE checklist assessed tele-facilitator’s ability to use the videoconferencing application, identify parts of the equipment, placement of the transducers, connections of the equipment, and instruct the patient for the testing. The first and third author carried out the OSCE examination.

The labeling form with images of the equipment was used to assess the proficiency in labeling the components of the equipment. Correct responses received a score of one, while erroneous responses received a score of zero. The knowledge questionnaire, OSCE checklist, and labeling forms were validated for relevance, adequacy, and appropriateness by two audiologists (one with 3 years of experience and one with 14 years of experience in tele-practice) and an auditory verbal therapist with 15 years of experience.

#### Step 2: Tele-diagnostic audiological testing.

##### Patient site set-up:

**Fig 2:**
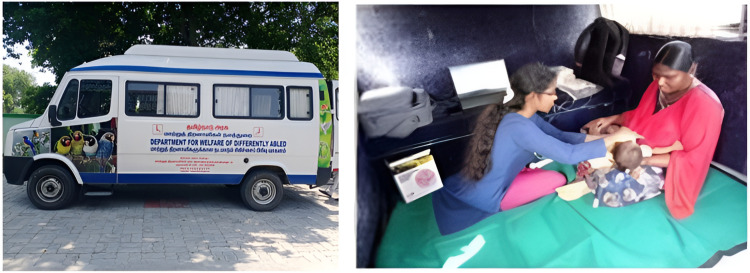
Set-up of the mobile-van.

The tele-diagnostic testing was conducted in a mobile-van near the Upgraded Primary Healthcare Center with 4G internet bandwidth enabled using a mobile phone hotspot. Internet was available through a few limited service providers in some of the locations, hence multiple service providers had to be explored. This van was equipped with a bed, fan, power sockets, and seating facilities. The mobile-van was scheduled to visit each Upgraded Primary Healthcare Center located at all 4 blocks once in a month on a fixed day of the week. The mobile-van set-up is shown in Fig 2.

The test equipment included video-otoscopy (Firefly Pro DE550), Pure Tone Audiometry (PTA) (Harp Inventis Plus calibrated to ANSI S3.6 standards), Oto-Acoustic Emission (OAE) calibrated as per EN 60645-6:2010 standards, and Auditory Brainstem Response (ABR) calibrated as per EN 60645-7:2010 standards (Neuro-Audio version 1.0.104.1) was used for the diagnostic assessment of hearing.

The laptop (Lenovo) was pre-installed with software for remote testing. The Teamviewer application (version 15.40.8) and V-See application (version 4.17.1) were installed on the laptop. A web camera was connected to the laptop for videoconferencing on the remote site. These features helped the audiologist to view the placement of transducers, otoscope, probe tip, insert tip and electrodes with more clarity.

##### Set-up at the audiologist site:

A laptop (Acer) with 8.00 GB RAM and 11thGen Intel(R) Core (TM) i5-1135G7@ 2.40 GHz was used by the audiologist at the University to provide tele-diagnostic evaluation and tele-rehabilitation services. The laptop was configured with Teamviewer application (version 15.40.8) and V-see application (version 4.17.1) for the audiologist to remotely access the laptop.

##### Testing method:

All children who required diagnostic testing either from the hearing screening using SRESHT screener or suspected of hearing loss by government pre-school teachers (anganwadi teachers), village health nurses, or community workers were asked to follow-up at the mobile van during the scheduled visit to the block level Upgraded Primary Healthcare Center, closest to the child’s home. The tele-facilitator provided instructions about the test prior to the test procedure. The remote audiologist clarified any doubts via videoconferencing. The audiologist used remote computing to conduct tele-testing in real-time for all test procedures, adhering to standard test protocols.

The audiologist counseled all children diagnosed with hearing loss to enroll for hearing aids/cochlear implants at the District Differently Abled Welfare Office, the district General Hospital, or other nearby government centers. The parents were also given the option to use temporary hearing aids (Tarang- 4 channel programmable affordable indigenous digital hearing aid) provided through the project and programmed according to their hearing loss, until they bought their own hearing aids/Cochlear implant. We emailed all the patient reports to the parents and also made them available to the District Differently Abled Welfare Office.

#### Step 3: Tele-rehabilitation.

A dedicated noise-free room was chosen in the SSA Block Resource Centers. The VSee video-conferencing application was installed on the laptop available at the respective Block Resource Centers. A computer assistant available at the center assisted with the process. The facilitator identified available therapy materials at the Block Resource Center for use during the sessions. The facilitator procured additional therapy materials or asked the parent to bring them for the session. Each therapy session lasted for 45 minutes.

#### Step 4: Parental satisfaction with tele-practice (diagnostic and rehabilitation).

To assess parental satisfaction with tele-diagnostic evaluation, a 10-item questionnaire with open and closed ended questions was used. Relevant items from an existing questionnaire available in Tamil was taken from Ramkumar et al. (2016) [35]. The tele-rehabilitation satisfaction questionnaire was modified and face validated in Tamil language from the one developed by Jahromi & Ahmadian (2018) [19]. This questionnaire had 11 questions (5 close-ended, 5 close-ended with 1 open choice and 1 open-ended question).

Both the satisfaction questionnaire’s (tele-diagnostic and tele-rehabilitation) relevance, adequacy, and appropriateness were face validated (see S5 file) by two audiologists and one auditory verbal therapist (AVT). Following the tele-diagnostic assessment, the tele-diagnostic satisfaction questionnaire was administered. The tele-rehabilitation satisfaction questionnaire was administered following two tele-rehabilitation sessions (see S6 file).

### Analysis

1. The tele-facilitator’s knowledge and skill adequacy were evaluated using the percentage of correct responses and the task completion of pre-defined skills, respectively.
2. The limited efficacy measure (from Bowen’s Feasibility Framework, 2009) [17] was used to evaluate outcomes of the pilot by comparing against the existing outcome data available prior to the commencement of the pilot. We analyzed the following outcomes within the context of limited efficacy:
  *i) Rate of follow-up for tele-diagnostic evaluation before and after the commencement of pilot:*
  The number of children with refer results in screening was compared with the number of children who followed-up for tele-diagnostic evaluation. We further compared this data with the number of children who underwent screening at the existing district General Hospital three months prior to the pilot’s commencement.
  *ii) Time lapse between screening and diagnosis of hearing impairment before and after the commencement of the pilot:*
  For the purpose of this analysis, the data of all children diagnosed with hearing loss via tele-diagnostic evaluation as part of the pilot (Group 1) was compared with equal number of children pre-diagnosed with hearing loss currently in Early Intervention Centre (Group 2). The parents of children enrolled in Early Intervention Centre were contacted, and details were collected from their records (screening date, place, and date of diagnosis). Due to the nature of the study, the information obtained was specific to the individual child who participated in the study and could be identified. For Group 1 and Group 2, the time interval between the screening/suspicion of hearing loss stage to diagnostic evaluation was calculated. We calculated the median for both Group 1 and Group 2 for comparison.
  *iii) Rate of follow-up for tele-rehabilitation before and after the commencement of the pilot:*
  The number of tele-rehabilitation sessions completed per child was documented and compared with the number of rehabilitation sessions before the pilot for a period of 3 months.
3. Patient/caregiver satisfaction with tele-practice measured using the questionnaire was analysed using percentage analysis and reported descriptively.

## Results

### Tele-facilitator knowledge and skill post training

Tele-facilitator’s knowledge and skill adequacy was a prerequisite for them to be involved in the pilot. In all, two facilitators underwent training. No knowledge/ skill adequacy assessment was done pre-training, as they were not expected to know any information pertaining to tele-practice.

On post training knowledge assessment, both trainees 1 and 2 obtained 90% correct responses, with accurate answers for questions related to remote-testing. The incorrect responses were for questions about how the facilitators will troubleshoot when any interruption arises during the audio/video call.

In the skill adequacy assessment, both trainees successfully executed 18 out of the 20 expected skills, including setting up an internet connection, positioning transducers, probes, and electrodes, and preparing the patient for each test. Trainee 1 was unable to turn on the video in the team-viewer application to establish video-conferencing, and trainee 2 had difficulties placing the bone vibrator for pure tone audiometry testing alone.

We asked the trainees to label various parts of the equipment/transducers to ensure they understood the audiologist’s instructions during remote testing. Trainee 1 obtained 47% correct responses, and trainee 2 achieved 93%. When asked to describe the utility of the part labeled, both trainees accurately described the usage of each part, even if they did not label it correctly. Both trainees were involved as tele-facilitators, while trainee 2 was assigned as the primary facilitator based on the trainees score of more than 80% on both knowledge and skill evaluation, trainee 1 was asked to shadow and support trainee 2.

### Limited- efficacy outcomes

#### Rate of follow-up for tele-diagnostic evaluation.

In all, 41 children (age range of 5 months to 5 years 3 months) were screened by nurses using the SRESHT screener at the four Upgraded Primary Healthcare Centers of the district. Of them, 11 (26.8%) had ‘refer’ results in the screening and 10 (90.9%) of them followed up for tele-diagnostic evaluation. A parent of one child was not willing to complete a confirmatory diagnostic hearing evaluation as she did not suspect/perceive any problem. Table 1 provides month-wise data on screening and follow-up post pilot. In addition, two children directly came for tele-diagnostic evaluation without undergoing hearing screening based on suspicion of hearing loss. We confirmed that 11 out of the 12 children had hearing loss.

**Table 1:** Screening and follow-up for tele-diagnostic testing for children with refer results post pilot.

Month and Year	Number of hearing screening (birth to 6 years)	Number of children with refer results	Number of children followed up for tele- diagnostic testing
April 2023	8	2	2
May 2023	32	9	8
June 2023	1	0	2*

*Directly came for tele-diagnosis.

For comparison, we collected month-wise data on the number of children screened at the district Early Diagnostic Centre in the General Hospital over a three month period from January 2023 to March 2023 (Table 2). An audiologist screened 459 children at birth using an OAE screener. There was only one referral from the screening, and this child received the diagnostic evaluation by traveling to a nearby city. Therefore, the number of children followed up for tele-diagnostic evaluation before the commencement of the pilot was one and after was 12.

**Table 2:** Screening and follow-up for diagnostic testing for children with refer results pre pilot.

Month and Year	Number of hearing screening at birth	Number of children with refer results	Number of children followed up for diagnostic evaluation at a nearby city
January 2023	176	0	0
February 2023	107	0	0
March 2023	176	1	1

An age-appropriate tele-diagnostic test battery was conducted to confirm hearing loss among 10 children who were referred from Upgraded Primary Health Centres and two other children who were referred directly from Early Diagnostic Centre at the General Hospital. Out of the 12 children who were tested, one child had normal hearing sensitivity, seven children had confirmed sensorineural hearing loss, two children with bilateral minimal to mild hearing loss and bilateral mild to moderate hearing loss were suspected with neuro-maturational delay and two children had conductive hearing loss. The demographic details, case history, test performed and the diagnosis of all the 12 children is tabulated (see Table 3).

**Table 3:** Demographic data, case history, tests completed and provisional diagnosis for tele-audiology diagnostic children.

Name	Age/ Gender	Case history	Tests completed	Test findings
Child 1	2 years/ Male	The parents suspected hearing loss for their child and did not report any significant birth/medical/family history. The child had a delayed speech and language development milestones and then underwent SRESHT screening. Based on the result from the screening they had followed up for tele-diagnostic evaluation.	ABR*	Bilateral profound hearing loss
Child 2	1 year/ Female	The parents suspected hearing loss for their child and did not report any significant birth/medical history. The child had a delayed speech and language development milestones and then underwent SRESHT screening. Based on the result from the screening they had followed up for tele-diagnostic evaluation.	ABR	Bilateral profound hearing loss
Child 3	3 years/ Female	The child had a refer result in the SRESHT screener. Previous audiological reports suggest bilateral middle ear pathology and recommended to follow up for detailed audiological evaluation but they did not follow-up. The child has been diagnosed with Angelman Prader Willi syndrome, where she presented with hypotonia, microcephaly and global developmental delay and currently attending physiotherapy. No significant birth/family history was reported. The child’s developmental milestone was delayed.	Video-otoscopy, OAE * , ABR,	Bilateral minimal to mild? conductive hearing loss.
Child 4	5 months/ Male	The child was brought from the adoption center with ‘refer’ result in the SRESHT screener and with a history of ear discharge in the left ear. The child did not have any significant birth/medical history. The child had undergone ABR testing at Trichy on 10/09/2023 and reports suggest that for right ear, the hearing sensitivity is within normal limits and for left ear the recording could not be completed as the child woke up.	OAE, ABR	Right ear: Minimal to mild hearing loss(?) Neuro-maturational delay Left ear: Minimal to mild hearing loss (?) Neuro-maturational delay? conductive hearing loss
Child 5	7 months/ Female	Child was brought as a part of routine assessment from an adoption centre followed by ‘pass’ result in the SRESHT screener. The child had no significant birth/medical/family history.	OAE	Indicative of normal outer hair cell functioning
Child 6	7 months/ Female	The child was brought from the adoption center with a ‘refer’ result in the SRESHT screener. The child had no significant birth/medical/family history reported. The child did not attain cooing at the age of 5 months. The child had a history of ear wax and usage of ear drops for 20 days in both ears. The child had undergone ABR testing at Trichy on 17/9/2023 and reports suggest that right ear is (?) Severe hearing loss and left ear the hearing sensitivity is within normal limits.	OAE, ABR	Bilateral mild to moderate hearing loss? Neuro- maturational delay
Child 7	5 years/ Male	The child has been diagnosed with intellectual disability (ID) and presented with the complaint of not responding to name call. The child followed-up for tele-diagnostic evaluation after obtaining refer results in SRESHT screening	Video-otoscopy , OAE, ABR	Right ear: Minimal to mild hearing loss? Middle ear pathology Left ear: Hearing sensitivity within normal limits
Child 8	2.5 years/ Male	Parental concern of not responding to loud sounds. The child had a significant family history of first degree consanguinity. The child had previously undergone OAE screening at Perambalur GH at the 1.8 years of age and ‘refer’ result in the SRESHT screener. Later the child had followed up for tele-diagnostic evaluation.	OAE, ABR	Right ear: Profound hearing loss Left ear: Severe to profound hearing loss
Child 9	3.5 years/ Female	The child was presented with a concern of not responding to loud sounds with no significant birth/medical/family history reported with a ‘refer’ result in the SRESHT screener. Otoscopic examination was done on 27/5/2023 and report says TM intact. Later the child had come for tele-diagnostic evaluation following hearing screening.	OAE, ABR	Right ear: Severe to profound hearing loss Left ear: Moderately severe to severe hearing loss
Child 10	5 years/ Female	Parental concern and complaint from the school that the child is not responding to name call and soft sounds. The child is a case of post- lingual hearing loss as the parents reported that the child had difficulty in hearing only after the age of 3 years. No significant birth/family/medical history was reported. Otoscopic examination was done on 27/5/2023 and report says tympanic membrane intact. The child had a ‘refer’ result in the SRESHT screener and then came for tele-diagnostic evaluation following hearing screening.	OAE, ABR	Bilateral severe to profound hearing loss
Child 11	6 years/ Male	The child presented with the complaint of not responding to loud sounds and not speaking age adequately. A significant family history of hearing loss was reported by the parent. The child did not undergo SRESHT screening and directly came for tele-diagnostic evaluation.	PTA * , OAE, ABR	Bilateral severe to profound hearing loss
Child 12	3.6 years/ Male	The child presented with a complaint of not responding to loud sounds and name call and reported with a history of forceps delivery during birth. No significant medical/family history was reported by the parent. The child did not undergo SRESHT screening and had directly followed up for tele-diagnostic evaluation.	OAE, ABR	Right ear: Moderately severe hearing loss Left ear: Severe to profound hearing loss

*OAE-Otoacoustic Emissions; ABR- Auditory Brainstem Response; PTA- Pure Tone Audiometry.

#### Time lapse between screening and diagnosis for hearing impairment before and after the commencement of the pilot.

**Fig 3:**
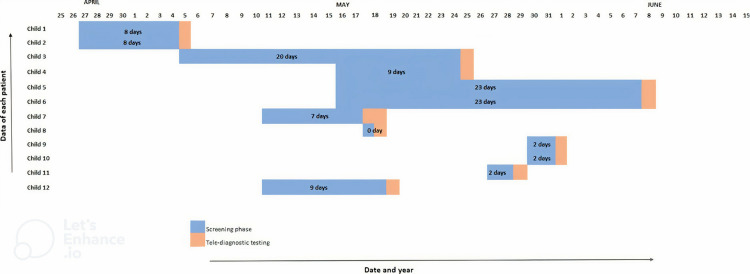
Time lapse between hearing loss suspicion/screening to tele-diagnosis.

**Fig 4:**
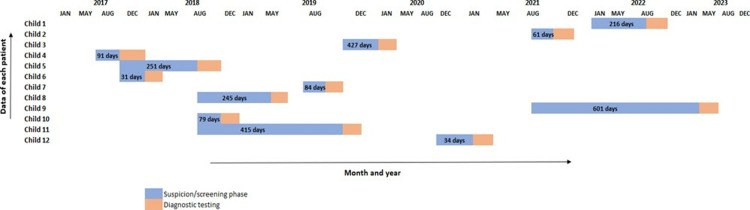
Time lapse between hearing loss suspicion/screening to diagnosis.

The time lapsed since suspicion/screening to diagnose a child with hearing impairment for Group 1 (post-pilot) and Group 2 (pre- pilot) are represented in Figs 3 and 4 respectively. The median number of days lapsed pre- pilot and post -pilot was calculated for both groups. The median time taken to diagnose a child for hearing using tele-practice (Group 1) was eight days, whereas pre- pilot, the time taken was 216 days (Group 2).

#### Rate of follow-up for tele-rehabilitation before and after the commencement of the pilot.

In all, five children attended tele-rehabilitation at the SSA-Block Resource Center. Four of these children were cochlear implant users who received special education at the Early Intervention Centre. Another child, identified from the pilot implementation, was directly enrolled in tele-rehabilitation using temporary hearing aids provided by the project. Each session was carried out for 45 minutes, where the initial 10 minutes were to review with the parent regarding home plan activities. At the end of each session, the goals and activities were reiterated to the parents and discussed any doubts/concerns regarding the child’s progress. Table 4 tabulates the number of sessions attended for a period of 3 months before and after the pilot’s commencement. The median number of sessions pre- pilot was zero and post- pilot was two.

**Table 4:** Rate of follow-up for tele-rehabilitation before and after the commencement of the test bed (refer the number given for child from Fig 3).

Child	Age/ gender	Case history	No. of sessions pre- test bed for a period of 3 months	No. of session post - test bed for a period of 3 months
Child 1	4 years/ Female	The child was diagnosed with hearing loss at 2.8 years of age in Trichy and procured CI at the age of 3.5 years of age.	0 session	3 sessions
Child 2	5 years/ Female	The child was diagnosed with hearing loss at the age of 3.2 years and had procured CI at the age 4.1 years at Coimbatore	0 session	8sessions
Child 3	6 years/ Male	The child had undergone diagnostic evaluation at the age of 3.2 years and procured CI at the age of 4.1 years in Chennai.	0 session	2 sessions
Child 7	4.6 years/ Male	The child had diagnostic evaluation at the age of 1.8 years and procured CI at the age of 2.6 years in Chennai.	0 sessions	1 session
Child 9	4.6 years/ Male	The child had undergone diagnostic evaluation at the age of 3.5 years and had repeated the hearing testing in Trichy as they had lost the previous reports. Initially they had been using the temporary hearing aids (Tarang) and later had procured their own hearing aid.	0 session	1 session

CI-Cochlear Implant

### Patient/ caregiver satisfaction with tele-practice

#### Tele-audiology diagnostic service.

All parents (n = 12) of children who underwent tele-diagnostic testing completed the satisfaction questionnaire. Parents reported to have had either a ‘good’ (58%) or ‘very good’ (42%) experience, and none of them reported to have any hesitation to avail tele-testing. All parents (100%) reported being able to comprehend and express their concerns with the audiologist in a satisfactory manner.

The tele-diagnostic testing was conducted in the mobile-van for 50% of the children, for 33% testing was shifted from mobile-van to a room within the Upgraded Primary Healthcare Center due to poor ventilation. Few parents (17%) reported that during the initial visit in the mobile- van the test was not completed as the child was not cooperative and they had to follow-up to the Early Intervention Centre for testing.

The majority (92%) of parents did not require assistance from the tele-facilitators in asking their questions to the audiologist. However, few (8%) of them required some assistance from the tele-facilitators. The majority of the parents (75%) reported having had no difficulty during the tele-testing. Parents reported that in the absence of such tele-testing service they would have travelled to nearby cities such as, Trichy (60 km) (66%), Chennai (276 km) (25%) and few (8%) planned to check if services were available at the district General Hospital in Perambalur. The majority (75%) of them preferred that such a testing alternative be continued in the district, while some (25%) parents preferred to undergo hearing testing in-person in nearby cities.

#### Tele-rehabilitation services.

All parents were satisfied (80%) with tele-rehabilitation since the clarity of speech with the therapist was adequate via videoconferencing. They were all ‘satisfied’ (60%) in communicating with the therapist and had ‘met their expectation’ (80%). Parents found it easy (80%) to voice out their concerns and all the parents (100%) said they would recommend tele-therapy to others, and were ‘very comfortable’ (80%) as the location was suitable for all the parents. Majority of the parents were ‘very likely’ (60%) to continue with this service as they were able to recognize the instructions given by the therapist (60%) and also trusted the therapist “very well’ (80%).

## Discussion

This study explored the pilot outcomes of the first known effort to integrate tele- practice services for childhood hearing loss in the public health system of a low middle income country [34]. Very limited studies have explored the outcomes of providing comprehensive hearing care using tele-practice [7,14,15].

Tele-facilitators in audiological testing are required at the patient site to support placement of probes, electrodes, and transducers as required and also provide technical ftcplosort [20]. In a scoping review, it was identified that predominantly audiologists, audiology students, and unspecified technicians served as tele-facilitators and limited studies reported details of the training they had received [21]. In the current study, we trained a special educator, deputed from the government Early Intervention Centre, to support remote testing as needed. Such capacity building is necessary to optimize shared resources to support implementation of services where none exist. We also trained a second trainee special educator as a backup to prevent service discontinuity due to absences.

The comprehensive model of tele-practice was conceptualized as a community-based model that includes children from birth to 6 years, to overlap with the Government of India’s age criteria for early identification. The SRESHT screener supports the triaging of 40% (60 dB HL) hearing loss from the community to the nearest block level Upgraded Primary Health Centre for tele-diagnosis. However, due to the lack of diagnostic services in the district General Hospital, there was considerable demand for diagnostic services among parents and caregivers who had suspicions about their child’s hearing.

On the other hand, district General Hospital had catered only to the ‘at birth’ population using the OAE screener, which is capable of triaging even milder hearing losses (>25-30 dB HL). While the coverage was higher at birth, older children with hearing loss were unidentified and missed. In low-middle income countries, the age of identification of hearing loss still continues to be higher [22] and there are only a few screening programs that have focused beyond the newborn stage.

The comprehensive tele-practice model integrates screening at a primary health facility to facilitate block-level triaging of individuals requiring diagnostic testing. However, during the test bed, it was noted that, the suspicion and referrals were predominantly from the SSA special educators (educational department) and then were redirected to the Upgraded Primary Health Center for screening. Therefore, screening at the SSA- Block Resource Center can also be explored to assess if triaging is better compared to Upgraded Primary Healthcare Center- based screening.

Despite the use of a test battery approach, the children underwent only the minimum required tests to diagnose their ear and hearing condition. We optimized the use of resources like mobile van availability, internet bandwidth, and limited time to obtain an accurate diagnosis. Sometimes, some tests were not conducted if children were less co-operative.

Even though the follow-up rate at the district General Hospital was 100% in comparison to 90.8% for tele-diagnosis, it should be noted that only one child needed diagnostic assessment in the former. Therefore, this may not be a true indication of the follow-up compliance. The loss-to-follow-up rate for tele-diagnostic testing was 9% in the current study, which is much lower than that reported in several EHDI programs in India [23–27].Lower loss to follow-up is also reported in few other studies that implemented tele-diagnostics in EHDI in Canada [14] and the USA [7].This suggests that in a tele- practice model the loss to follow-up rate is less when compared to in-person testing.

We found that the time between the suspicion of hearing loss/screening to diagnosis was less for the children who availed tele-diagnostic testing. This confirms that having local resources within a block enables better follow-up and timely diagnosis. Similar results were found earlier in a study where the time taken between second screening and tele-ABR assessment was less in a rural community-based program with a median interval of 30 days [29].

The findings suggest the potential for improvement in the rate of follow-up for tele-rehabilitation but do not assert it, owing to the small sample. Improvement in the number of sessions attended was also reported in a tele- auditory verbal therapy program in Logan, USA [28]. The coordinated follow-up and scheduling by the tele-facilitators and the therapist based on the availability of the parent/caregiver is likely to have promoted better follow-up.

The majority of the parents in the current study did not have any difficulty during the testing, but some reported poor ventilation in the mobile-van which caused difficulties for the child to be comfortable and cooperative for the testing. While most parents preferred tele-testing within their block, a few expressed a preference for in-person diagnostic services in nearby cities. Even though during COVID-19 there was a surge in tele-practice across all health disciplines, there exists a preference for in-person services [30,31]. Therefore, tele-practice can augment services where none exist and until suitable infrastructure and resources are allocated.

The current study also suggests that caregivers found the tele-rehabilitation to be acceptable, trustworthy, and the location (at block level) to be suitable, comfortable, satisfied with the level of communication with the therapist, and had met their expectations. As they had trust in the clinician, they were also likely/very likely to continue receiving tele-rehabilitation services. In another study too, high satisfaction was reported for tele-consultations for pediatric health [33].Trust in audio-video communication was reported by majority of the patients, which is likely to have resulted in better satisfaction [19]. These results are found to be similar with another study [32], where majority of them felt that tele-therapy was convenient, cost-effective and improved the attendance of the sessions. But some had reported about the increased use of gadgets and screen time for their children.

There are limitations to our current findings, owing to small and convenient sample size (due to test-bed), as well as the limitations of a questionnaire-based approach that does not provide an in-depth, unbiased perspective. These will however be explored on an ongoing basis to study the feasibility outcomes of the overall implementation in the future.

## Conclusion

Overall, it can be concluded that this comprehensive tele-practice model can be effectively implemented to overcome professional shortages, and was beneficial in reducing time gap between screening/suspicion and diagnosis and also enhanced attendance for rehabilitation. The pilot helped to identify the aspects of the model that are suitable (e.g., block level implementation of diagnostic and rehabilitation services, community level screening including older children) and the aspects that need adaptations (ventilation in the mobile van, center-based tele-testing within the block, multi-adapter internet dongles for more stable internet, or a mobile-phone call for audio communication, with videoconferencing alone through the desktop-based software). This project is on-going, and suitable adaptations are made based on the findings of the pilot. The feasibility outcomes of implementing this model will be studied using the limited-efficacy measures, economic analysis, as well as qualitative methods to assess the various other constructs of Bowen’s framework.

## Supporting Information

S1 Data Procedure Manual for telefacilitator training. (PDF)

S2 Data Knowledge questionnaire. (DOCX)

S3 Data Skill set checklist. (DOCX)

S4 Data Label parts of equipment. (DOCX)

S5 Data Tele-diagnostic satisfaction questionnaire. (DOCX)

S6 Data Tele-rehabilitation satisfaction questionnaire. (DOCX)
