# Supplementary material for: Pilot implementation outcomes of a community-based tele- practice model for identification and rehabilitation of children with hearing loss within a public-health system of a Rural District in Southern India
Source: PLoS One. 2025 Mar 19;20(3):e0319109. doi: 10.1371/journal.pone.0319109 (PMC11922231; doi:10.1371/journal.pone.0319109)
Supplement: S1 Data — (PDF) [file pone.0319109.s001.pdf]

PROCEDURAL MANUAL FOR TELE-FACILITATOR TRAINING IN ENGLISH

PROCEDURE MANUAL FOR TELE- FACILITATOR TRAINING

*This manual is developed as a part of an implementation project by The **S**ervices to**RE**ach children with **S**peech & **H**earing disorders using **T**ele-practice (SRESHT) (A SRIHER-India Alliance lab) in collaboration with the State Commissionerate for the Welfare of Differently, Tamil Nadu.*

TABLE OF CONTENTS

1. What is tele- testing for hearing/speech-language disorders?
2. Who is a facilitator and what are their roles?
3. Where is tele-testing proposed to be conducted in the community
4. About the manual
5. Steps involved in tele-testing facilitation in hearing/speech language diagnosisand rehabilitation

- What is needed for tele-audiology/hearing testing?
- Connecting to the audiologist/speech therapist via the video-conferencingteam viewer
- Connecting testing equipment
    - Tele Video-otoscopy
        - Instructions to the facilitator
    - Tele-Pure Tone Audiometry (PTA) and Speech audiometry
        - Instructions to the facilitator
    - Tele-Oto- Acoustic Emissions (OAE)
        - Instructions to the facilitator
    - Tele-Auditory Brainstem Response (ABR)
        - Instructions to the facilitator
    - Tele-speech language / Tele-rehabilitation
        - Instructions to facilitator

1. What is tele- testing for hearing /speech-language disorders?

Tele-testing for hearing/speech language disorders is done for a child/adult who is in a centre or location where there are no audiologists. The testing is done remotely, with the help of a facilitator. The patient will be at the mobile tele-van and the clinician will perform the testing from the institution.

A dedicated qualified audiologist and speech language therapist (ASLP) will perform the hearing and speech diagnostic testing from a hospital (e.g. SRESHT Lab at Sri Ramachandra Institute of Higher Education and Research), whereas the trained facilitator special educator or technician will be at the other end (study site) to assist the ASLP.

Tele-testing is equal in standards to in-person services, provided all testing standards are maintained and will be beneficial to patients in remote rural areas , where they cannot travel to cities for testing due to non-availability of testing professionals/ equipments in that area.

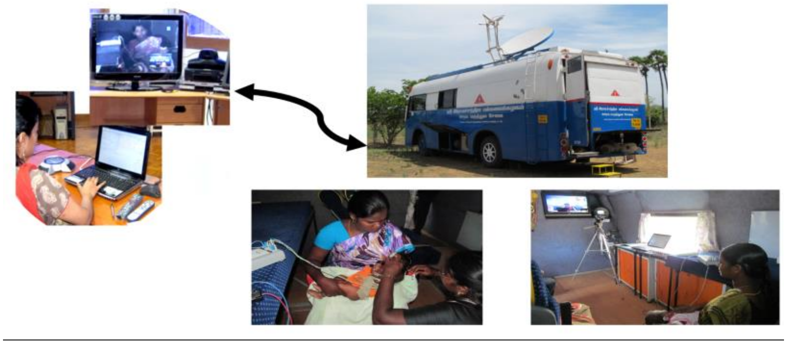

Figure 1: Tele-auditory brainstem response done in a mobile van at Thirukazhakundram block, Kanchipuram District with help of community worker as facilitator

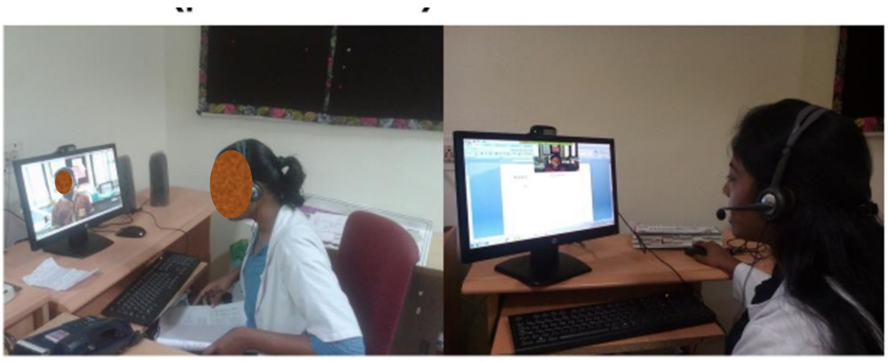

Figure 2: Tele-speech language assessment and rehabilitation from hospital by speech language therapist

2. Who is a facilitator and what are their roles?

Facilitator is a personnel available at the client's location to assist the professional in delivering appropriate clinical services remotely. Facilitator executes their roles and

responsibilities only under the guidance and supervision of a qualified professional (ISHA, 2020).

Caution: For ethical and legal reasons, the facilitator cannot conduct ANY audiologist or speech-language assessments without the supervision of an Audiologist or speech language therapist.

3. Where is tele-testing proposed to be conducted in the community

The DDAWO office has a mobile-van which will be enabled for tele-hearing and speech language diagnostic and therapeutic services.

This mobile-van will travel to each block and be available near the upgraded Primary health center/ Block Health Centre/ Block Resource Centre to provide diagnostic testing to those children who 'DO NOT PASS' hearing and or speech language screening conducted by community workers/ nurses.

4. About the manual

This manual is intended as a resource material that the facilitator can use post-training. The manual explains the remote testing site set-up along with details about audiological test procedures. The tasks associated with tele-hearing and speech language testing facilitation which is required in the mobile-van is provided in detail. Along with this manual, video demonstration for each of the testing procedures is also made available for your reference.

5. Steps involved in tele-testing facilitation in hearing / speech language diagnosis and rehabilitation

i) What is needed for tele-audiology / hearing testing?

In the mobile-van:

1. All audiology testing equipment and accessories
2. Laptop with all audiology test equipment's software pre-installed
3. Internet connectivity
4. TeamViewer app for video-call and remote control of equipment

5. Microphone
6. Speaker
7. Web-camera (built-in)
8. Facilitator
9. Parent or caregiver

Please make sure the following things are checked before starting the test procedure.

1. Ensure the laptop is fully charged.
2. Check whether the lights and fan in the tele-van are working
3. Ensure the surrounding environment is quiet for the testing
4. Check whether the charging plug points in the tele-van are working.

In the hospital site:

The audiologist / Speech language pathologist will check availability of the following;

1. Facilitator
2. Laptop with internet
3. Teamviewer App all for video-call and remote control of equipment
4. Microphone
5. Speaker
6. Webcamera (built-in)

ii) Connecting to the audiologist/speech therapist via the video-conferencing team viewer:

1. Switch on the laptop by providing appropriate password
2. Enable the internet connection in the laptop by clicking the internet icon and entering the password for wifi connection. Adequate internet connection is the key element for tele-practice.
3. Team viewer is an end-to-end encrypted remote desktop application that will be used for our tele-audiological testing

4. Click on the team viewer icon in the laptop. The icon is shown in the picture below

TeamViewer

5. Once the app starts, an user ID (10 digit number) and password will be displayed on the screen

6. Take a picture of the user ID and password displayed on the screen and send it to the audiologist at the other end/or tell the user Id and password to the audiologist through phone call (example is given below)

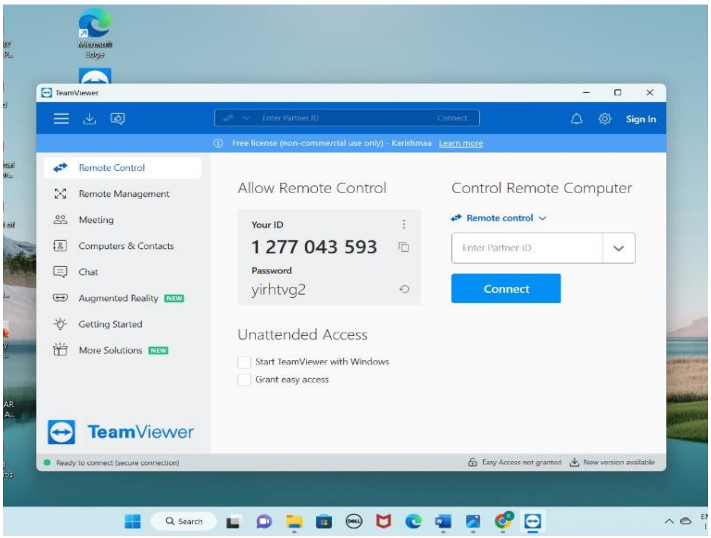

7. Once connected the audiologist at the other end will take over your laptop for remote operation and start the diagnostic testing.

8. During the testing, enable the video calling option available in the TeamViewer app and make sure your environment is quiet.

9. During conversation with the audiologist at the other end, enable the microphone and speaker option in the team viewer app.

iii) Connecting testing equipment:

Tele Video-otoscopy:

Video - otoscopy is a portable, handheld device used for examining the tympanic membrane and external ear canal to diagnose outer and middle ear pathologies. It consists of a camera with LED lights used to visualise inside the ear.

It has the following components

- Speculum of 3 sizes (3,45 mm)
- Otoscope with LED lights
- USB cable
- Magnifying lens

Components:

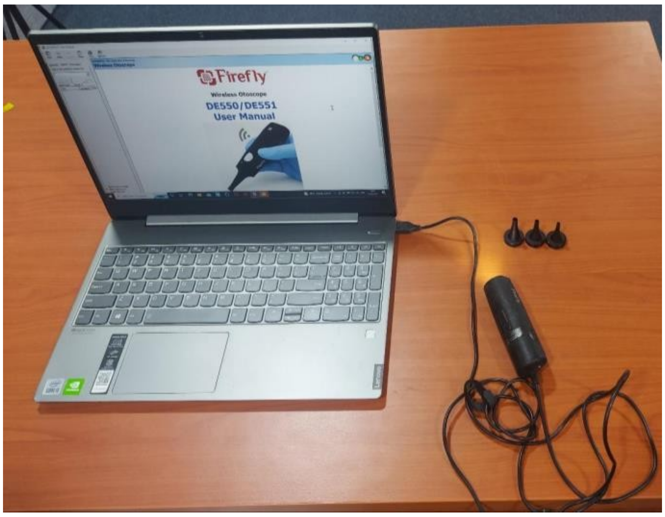

Usage of parts of the otoscope:

1.

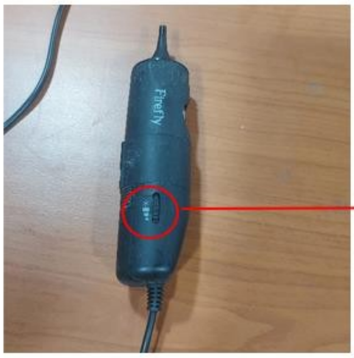

To turn on the light use this button

2.

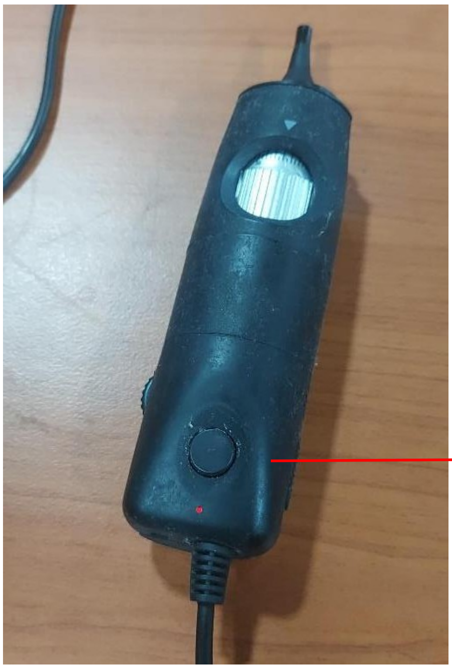

This button is used to capture images.

3.

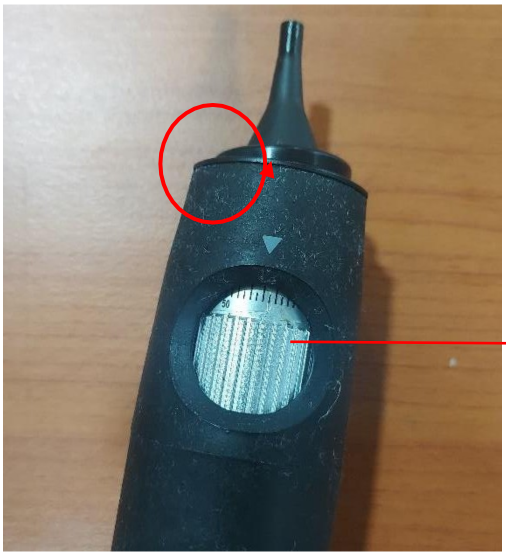

Magnifier- It is a scale to adjust the clarity of the image

5. Speculum are of 3 sizes: 3mm, 4mm and 5mm.The most commonly used size for children is 3 mm which is narrowest, if not we can use 4 mm size.

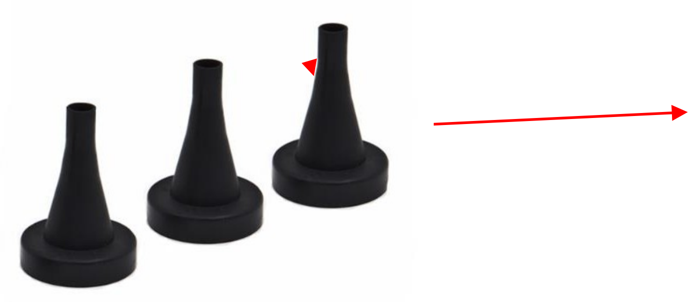

Speculum- This part is inserted into the ear based on the size of the child's ear to visualise the tympanic membrane

Instructions to the facilitator:

1. Connect the USB wire to the laptop

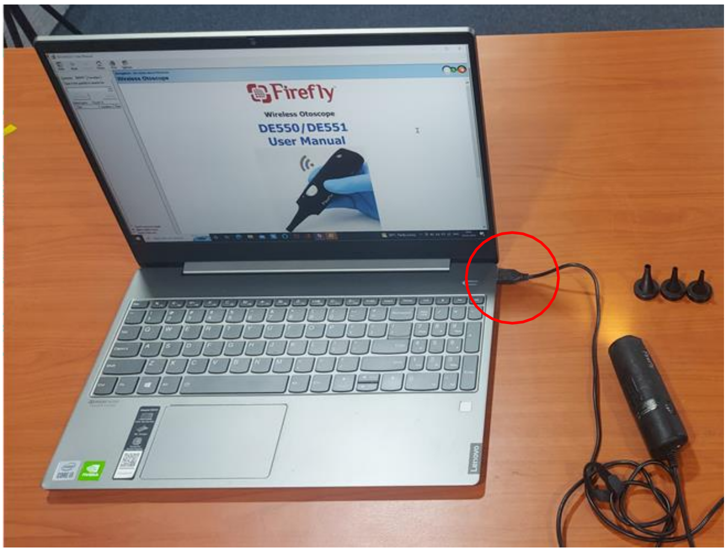

2. Connect to the audiologist using team viewer app as explained above

3. Turn on the light in the otoscope

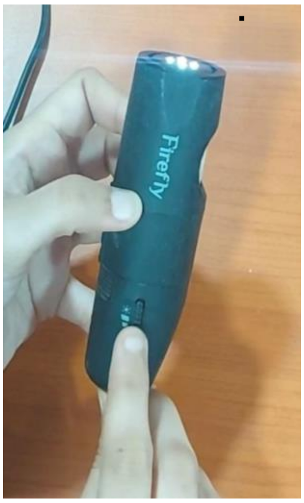

4. Insert the speculum into the otoscope

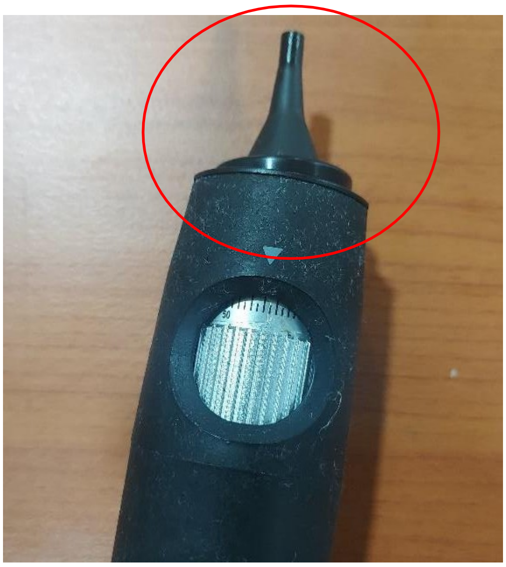

5. Place the otoscope into the ear.

For each ear there are different procedures to be followed which will be explained below.

For right ear – Use the left hand to pull the ear backwards and hold the otoscope like a pen in the right hand and insert it into the ear.

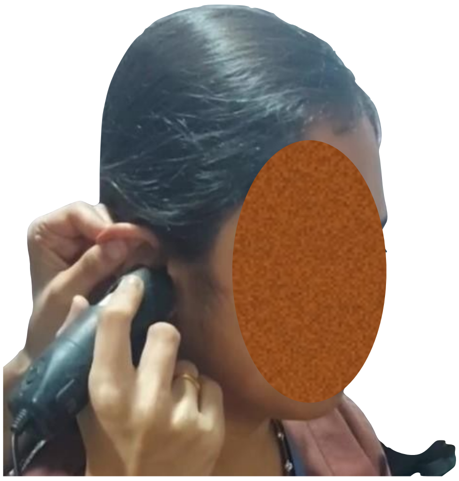

For left ear- Use the right hand to pull the ear backwards and hold the otoscope like a pen in the left hand and insert it into the ear.

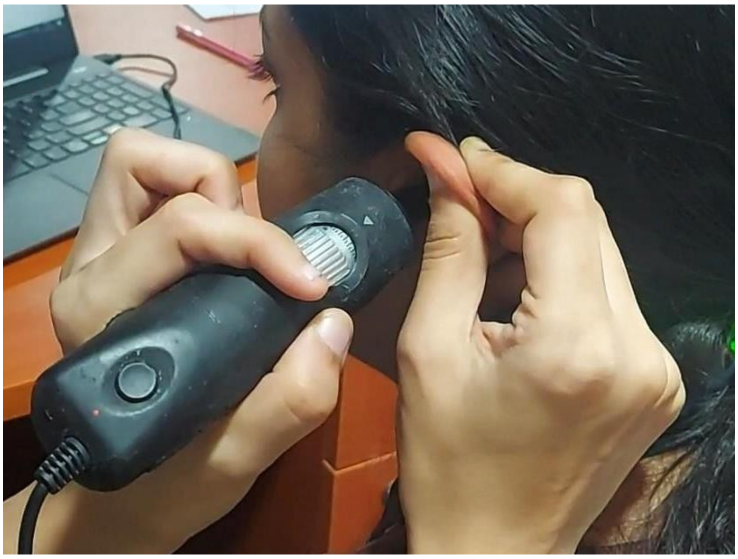

6. You will visualise the tympanic membrane from the laptop.

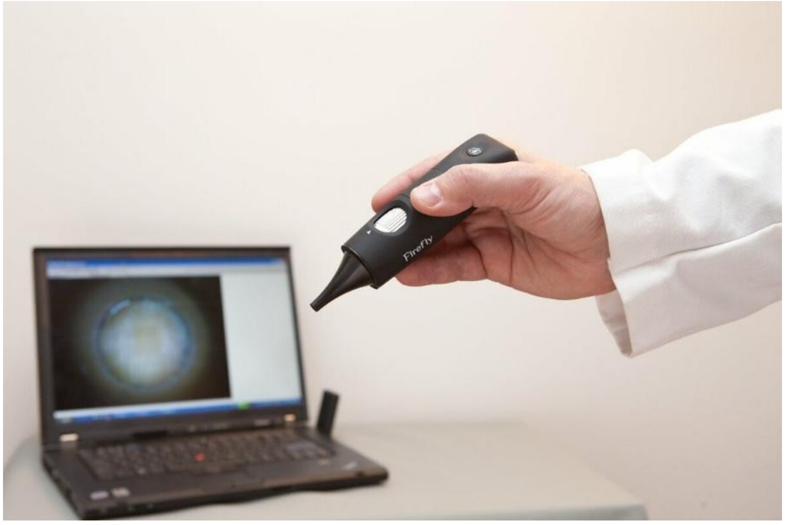

Tele- Pure tone audiometry (PTA) and speech audiometry:

Pure- tone audiometry (PTA) and speech audiometry is a subjective test used to assess the hearing level and to identify the word recognition ability of the child respectively. The test is performed on children with a cognitive age of four to six years

The components include: Audiometer (hardware), laptop (software), a USB cable to connect the audiometer to the laptop, headphones, bone vibrator and response button

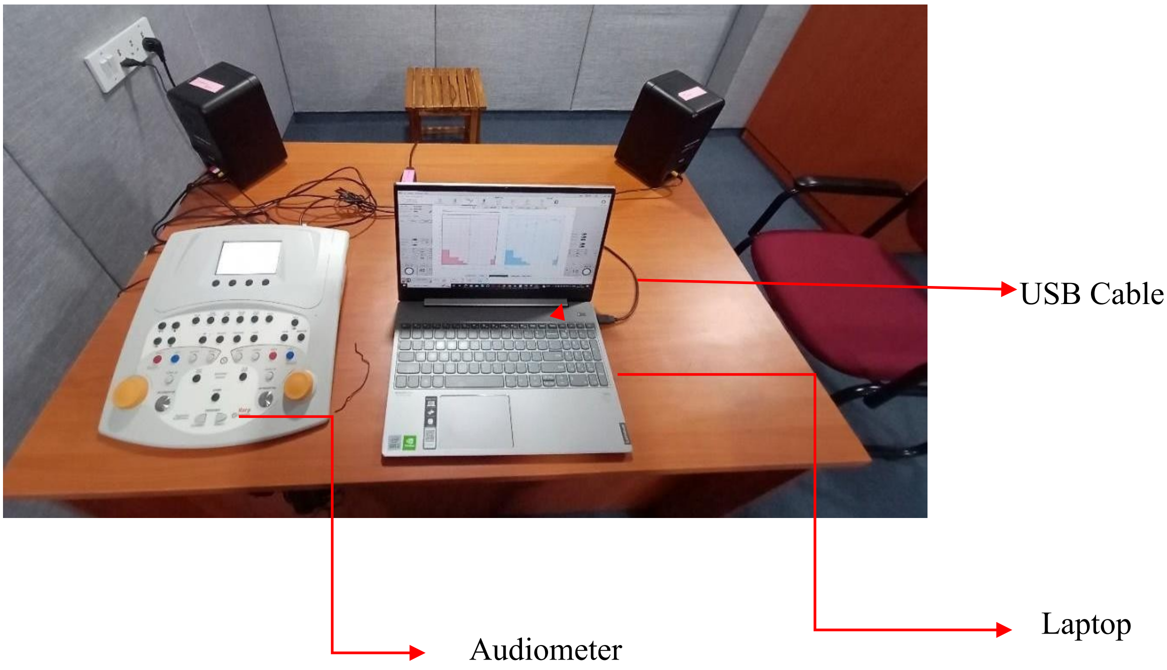

Instructions to the facilitator:

1. Connect the audiometer to the laptop through USB connector as shown in the above image.

2. After you have connected ‘PC controlled mode activated ‘will be displayed on the screen

3. Connect team viewer app as described earlier

4. Place the headphones onto the child’s ear. Make sure that right and left headphone placement is appropriate according to the colour (red colour for right ear, blue colourfor left ear).

For right ear- Red colour

For left ear- Blue colour

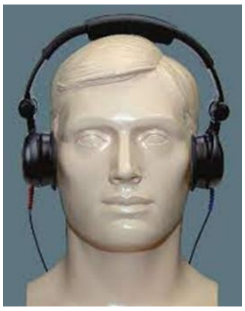

5. Adjust the size of the headphones according to the child's head (Refer video for better clarification)

6. Instruct the child/caregiver to respond by pressing the response button or by raising hand whenever the sound is heard.

"A sound will be heard from the headphones, it may be a loud sound or soft sound. First, the sound will be loud and then it becomes soft. Whenever you hear a sound press the response button or raise hand"

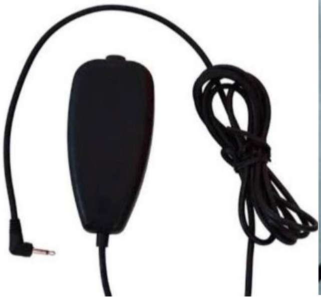

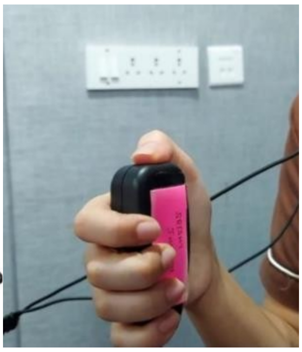

7. After completing both ears; place the bone vibrator behind the child's ear (mastoid bone). While placing the bone vibrator, adjust the hair at the site of placement. Instruct to the child/caregiver same as headphones.

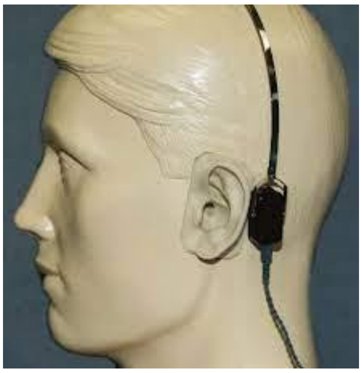

8. Then again place the headphone onto the ear and instruct the child to repeatthe words heard for speech audiometry testing

Tele-Otoacoustic emission (OAE):

Otoacoustic emissions (OAE) are used to assess the functioning of outer hair cells in the cochlea in response to low-level sounds. For this test, the environment must be quiet as this device is sensitive to noise

The child must be made to sit on a chair or lie down on the caregiver's lap in a comfortable position. Preferably, the testing is done while the child is sleeping. If the child is not asleep, we have to make sure that the child is quiet and does not shake the head during the testing

Components of OAE:

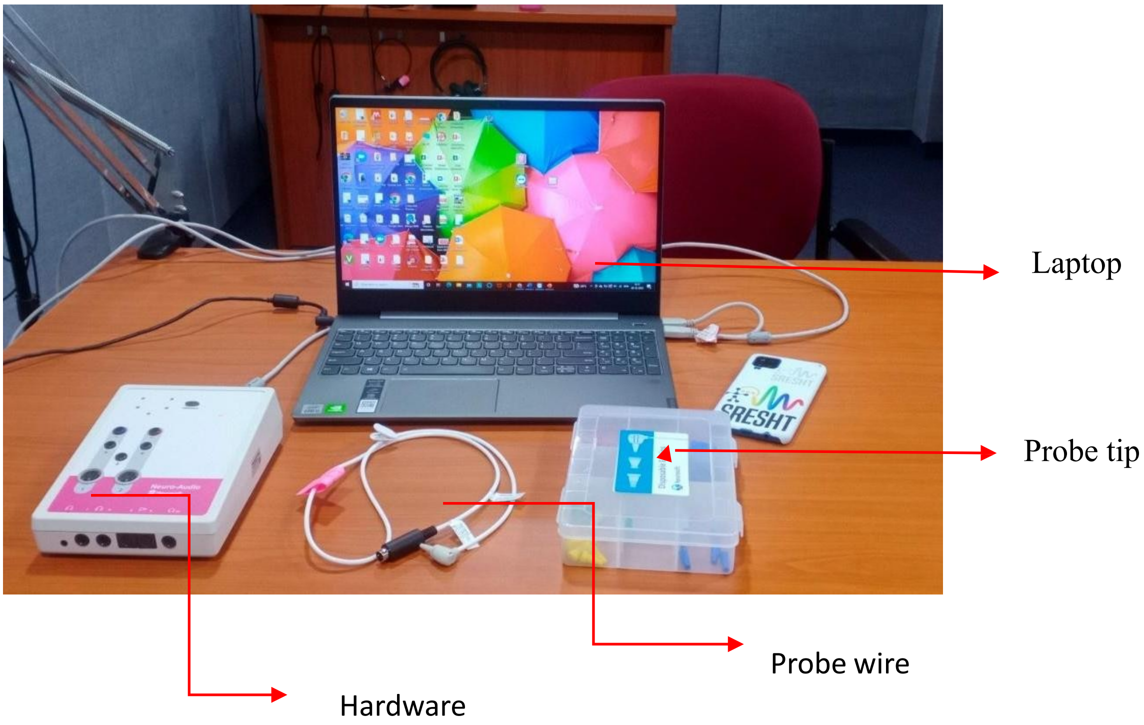

Instructions to the facilitator:

1. Connect the hardware to the laptop using the two USB cable, as shown in theimage below

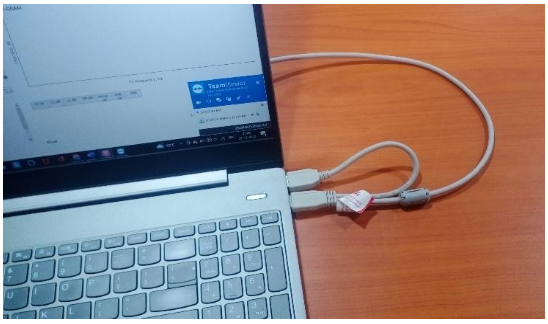

2. Connect to the team viewer app as described earlier.

3. Now the audiologist at the other end will be able to operate your laptopremotely.

4. Connect the OAE probe wire to the hardware as shown in the picture

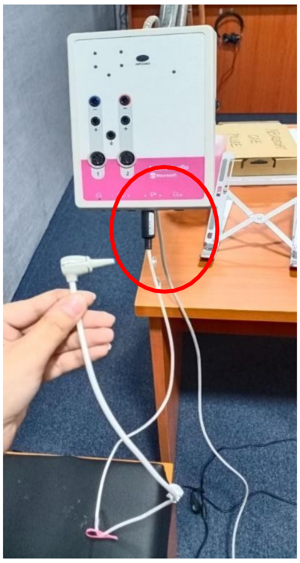

5. Select appropriate probe tip size according to the child’s ear canal.

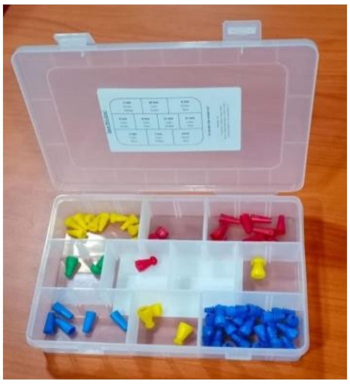

6. Place the probe tip as shown in the picture and then insert it into the child’sear canal by pulling the ear backwards

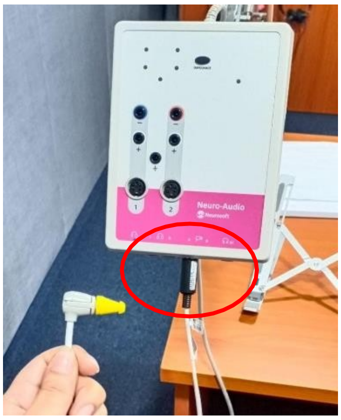

7. Then the audiologist will start the OAE testing

8. Then place the OAE probe to the other ear.

Tele- Auditory Brainstem Response:

Auditory Brainstem Response (ABR) is an testing to check the integrity of the pathway from the auditory nerve to the brainstem.

Instructions to the facilitator:

1. Connect to team viewer app as described earlier (Refer general instructions)
2. Make sure that the child is asleep for the testing
3. The surrounding environment should be quiet for the testing.
4. Keep the mobile phones away from the equipment to avoid electricalinterference.
5. Plug the insert wire to the hardware of the equipment as shown in the picture.

   In the letter R, insert the red colour wire; In

   the letter L, insert the blue colour wire

Insert wire **Hardware**

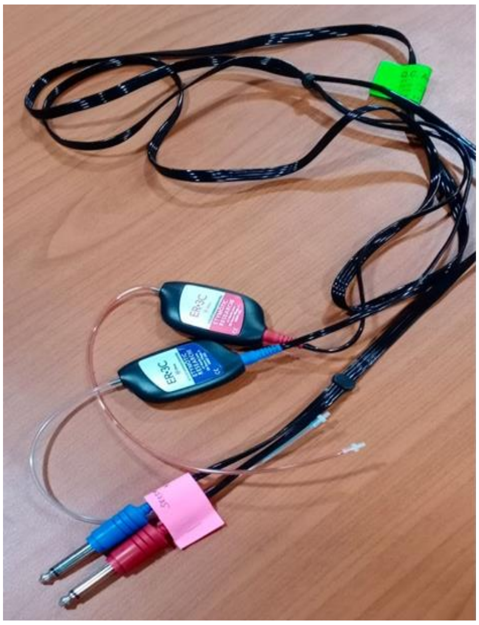

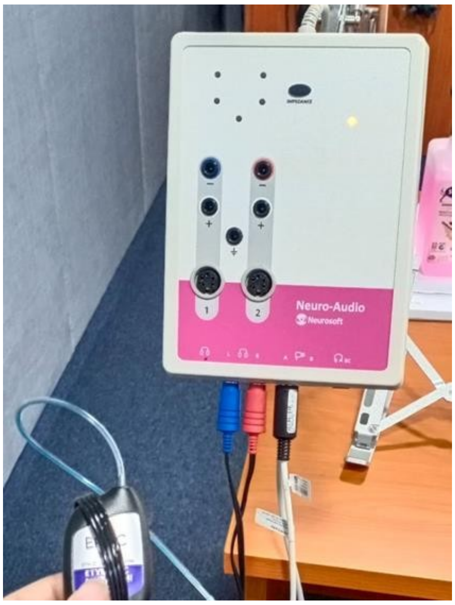

6. Select an insert tip according to the child's ear. If the child's age is less than18 months use the red colour tip or use the peach colour tip

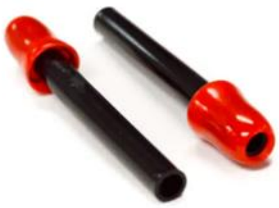

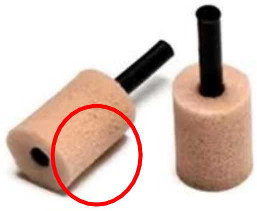

7. Place the insert tip into the white connector of the insert wire as shown in thepicture

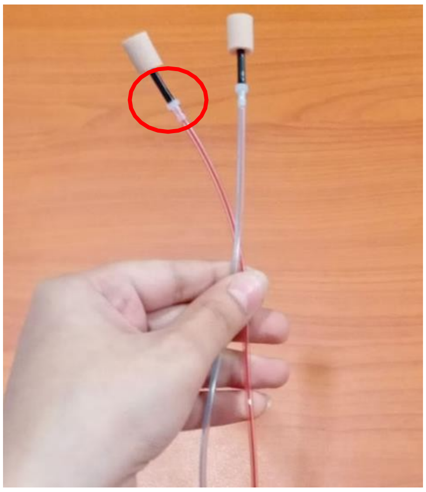

8. Insert the 4 electrode wires and jumper (white colour) into the hardware

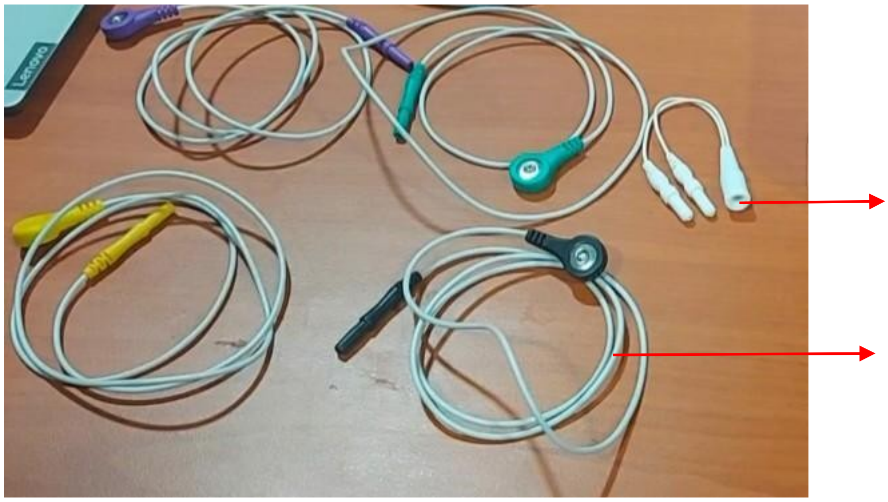

Jumper

Electrode wire

9. Insert the jumper in both the '+' symbols, and connect the other end of thejumper to an electrode wire.

   Insert any 2 electrodes wire into both the '-' symbols in the hardware. And

   insert the last electrode wire in the middle, which shows 3 lines.

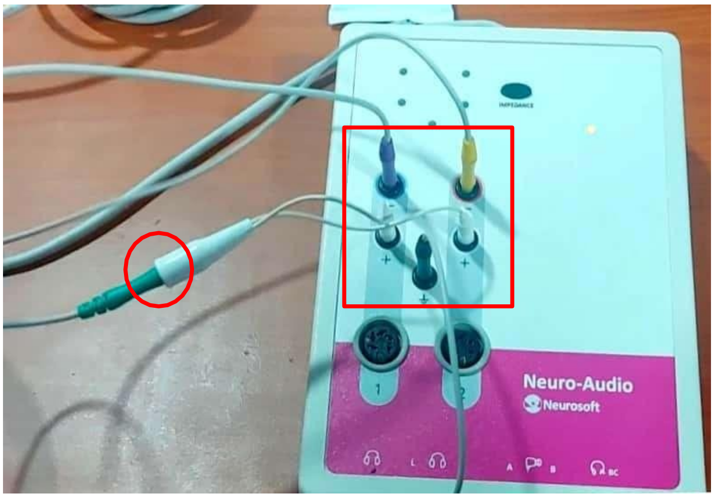

10. Plug in the electrode wires on button electrodes as shown in the picture

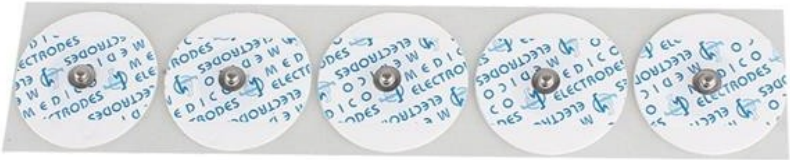

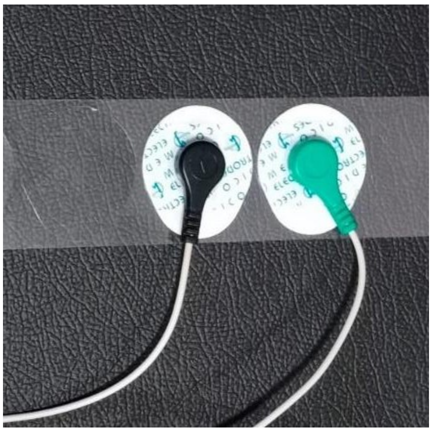

11. Clean the child's skin using Nuprep gel. Apply some Nuprep gel over the cotton and clean the upper forehead, lower forehead and both ear's mastoid bone properly (behind the ear).

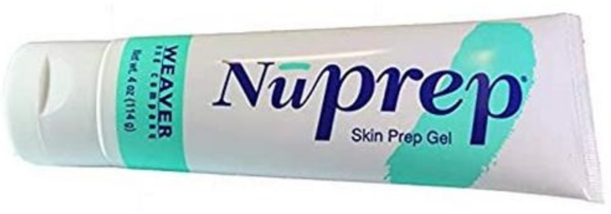

12. After cleaning the skin, place the button electrodes.

The '+' ve electrode wire should be placed on the upper forehead,

The Ground electrode wire which shows 3 lines on the hardware should be placed in the lower forehead

The two '-' ve electrodes should be placed in the bone behind the both ears.

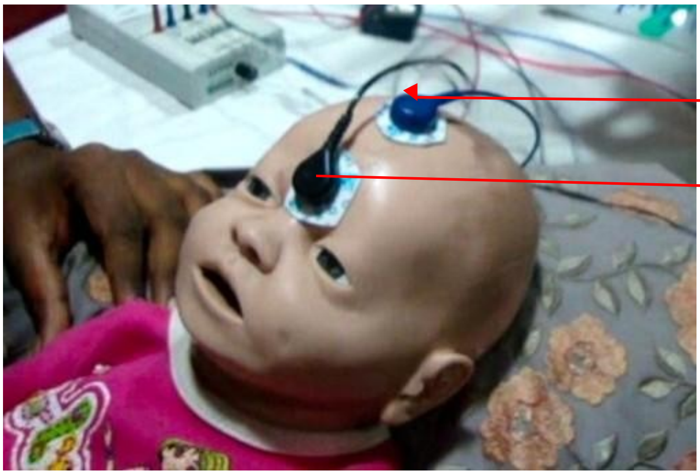

Upper forehead (+)

Lower forehead (electrode wire from the 3 lines shown in the hardware)

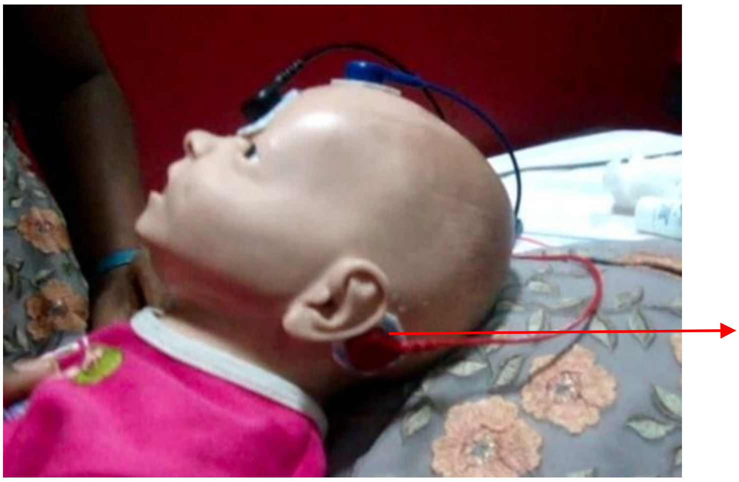

Bone behind the ear (-). The same placement is for the other ear also

13. Now place the insert tip into the child’s ear as shown in the picture below.

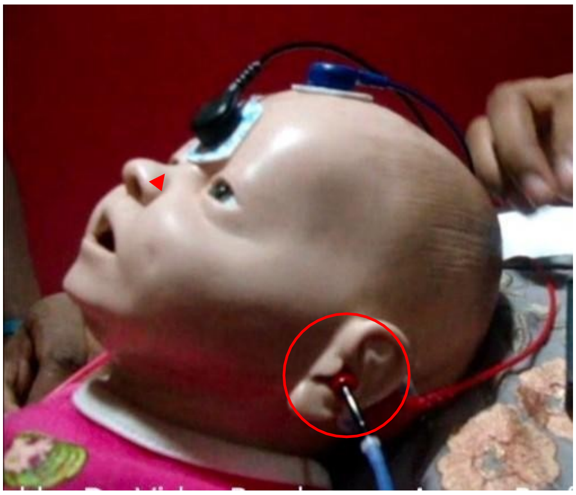

14. Ensure the wires are not tangled with each other.

15. After everything is set, the audiologist at the other end will start the testing and will provide further instructions according to the progress of the testing.

Tele- speech and language diagnostic assessment:

If the child had a ‘refer’ result in the screening, a detailed speech and language assessment will be conducted to identify the level of the child’s speech and language communication skills. Based on the results of the assessment, appropriate management plan will be formulated.

Instructions to the facilitator:

1. Open the V-see app in the laptop

2. Make sure that the video, microphone and speaker is enabled

3. Ensure that the room is well-lighted and quiet

4. Position the laptop in such a way that both the parent/caregiver and the childare visible to the Speech Language Pathologist.

5. Based on the child’s age and the complaint of the parent/caregiver, anappropriate assessment tool will be used.

6. The assessment materials are developed in digitised version.

Tele-rehabilitation:

Tele-rehabilitation is a therapeutic intervention to facilitate the child to improve the speech and language communication skills.

V. see app will be used for providing speech, language and hearing therapy

Pre-therapy assessment for audiological rehabilitation

Aural rehabilitation is a therapeutic intervention on a one-to one basis, given for individuals with who are fitted with Hearing aids (HA) or cochlear implant (CI) and it is focused on developing listening, spoken language communication.

VSee app will be used for the assessment procedures

The goals of this therapy program is to facilitate the child to learn and engage in meaningful conversation, to be assimilated into regular school programs and to have educational, social and vocational choices throughput the life.

Before providing therapy, we have to assess the current level of the child's language and communication skills.

Based on the results of the assessment, the goals of the therapy will be formulated

Pre-therapy assessment for speech and language rehabilitation:

Speech and language rehabilitation is an therapeutic approach which mainly focuses on improving communication of a child or to treat any speech disorders like stuttering.

First, we have to identify the current language or cognitive age of the child and a set of goals for this therapy program will be formulated accordingly.

Steps to conduct the testing:

1. Sit in front of bright lighting

2. Make sure the V-see app is installed in your laptop

3. Ensure that you have an adequate internet connection

4. Make sure that the child and the parent or caregiver is visible

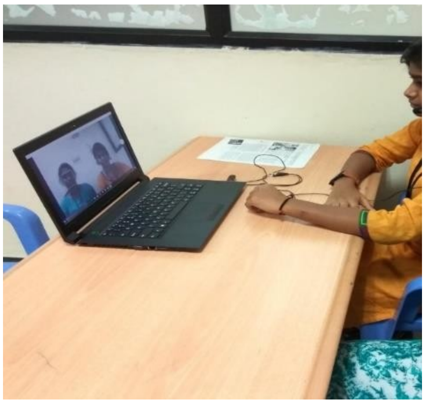

5. Make sure nobody enters the van while therapy is being given

6. Put all your phones in silent mode

The following materials will be used and shall be prepared by the parents/caregiver prior to the session.

1. Vehicles (car, bus, etc)
2. Ball
3. Clay
4. Animal toys
5. Baby doll
6. Building blocks
7. Stack ring
8. Photos of family members
9. Vegetable and fruits set
10. Cooking toys
11. Story books (if any)
12. Reinforcement toys (star sticker, smiley sticker)
13. Flash cards

This manual was developed as a part of the project '***Effectiveness of a comprehensive tele-practice model for identification and rehabilitation for children with hearing and speech - language disorders in rural communities***' by:

Ms. Karishmaa C

M.Sc. Audiology

Sri Ramachandra Institute of Higher Education &

Research (DU), Porur, Chennai -116

Professor. Vidya Ramkumar

DBT/Wellcome Trust India Alliance,

Intermediate fellow in clinical and

public health, Faculty of Audiology

and Speech Language Pathology,

Sri Ramachandra Institute of Higher Education &

Research (DU), Porur, Chennai -116.

<u>To contact us:</u>

Email id- sresht.sriher.ia@sriramachandra.edu.in

Phone number - 044- 4592 839
