# Supplementary material for: Pilot implementation outcomes of a community-based tele- practice model for identification and rehabilitation of children with hearing loss within a public-health system of a Rural District in Southern India
Source: PLoS One. 2025 Mar 19;20(3):e0319109. doi: 10.1371/journal.pone.0319109 (PMC11922231; doi:10.1371/journal.pone.0319109)
Supplement: S2 Data — (DOCX) [file pone.0319109.s002.docx]

**KNOWLEDGE QUESTIONNAIRE**

**Name:**

**Age/sex:**

1. **A tele-hearing/ speech language testing facilitator:**
   1. Assists audiologists/ speech therapist in conducting remote hearing testing under the supervision
   2. Conducts hearing testing independently in places where there are no audiologists
   3. Helps in referral to GH/ DDAWO office
   4. Don't know
2. **When a child has to be tested for hearing by an audiologist from SRESHT at Sri Ramachandra Hospital, what will you do? Order the following:**
   1. Connect laptop to internet
   2. Open the team viewer app in the laptop
   3. Make a video call to the audiologist using the team viewer app.
   4. Connect to Team viewer app by telling the user id and password to the audiologist
3. **What is the name of the wire that connects the equipment to the laptop?**
   1. Probe wire
   2. USB wire
   3. Charger
   4. Don’t know
4. **The audiologist and Speech Language and Pathologist says “I cannot hear you” but you can hear the audiologist. In this case what will you do?**
   1. Check whether your microphone is ‘ON’
   2. Switch OFF the laptop and switch it ON again
   3. Check internet connection
   4. Don’t know
5. **The voice and video is interrupted/not clear during the video-call. What will you do in this case?**
   1. Check internet connection
   2. Close the video and see if interruption reduces
   3. Use phone call/WhatsApp video call to the ASLP till interruption reduces or resolves
   4. All of the above
6. **What will you do if the child has got up/ starts crying during the ABR testing?**
   1. Switch OFF the lights in the van to facilitate the child to sleep
   2. Turn OFF the van
   3. Both a and b
   4. Don’t know
7. **What will you do, if the ASLP says that video-otoscopy image is unclear?**
   1. Use a different speculum size
   2. Utilise the magnifier
   3. Both a and b
   4. Don’t know
8. **How will you connect to the Audiologist and speech language therapist for tele- rehabilitation?**
   1. Team viewer app
   2. V see app
   3. Whatsapp video call
   4. Don’t know
9. **When there is a delay between you and the audiologist and speech language pathologist or when the testing gets slow, it indicates;**
   1. Inappropriate usage of laptop
   2. Insufficient internet connections
   3. Incorrect connections
   4. Don’t know
10. **What will you do, if the internet connection gets disconnected in the middle of tele-testing?**
    1. Abort the testing
    2. Continue the testing on your own
    3. Call the Audiologist and Speech Language Pathologist immediately
    4. Don’t know
