# Supplementary material for: Pilot implementation outcomes of a community-based tele- practice model for identification and rehabilitation of children with hearing loss within a public-health system of a Rural District in Southern India
Source: PLoS One. 2025 Mar 19;20(3):e0319109. doi: 10.1371/journal.pone.0319109 (PMC11922231; doi:10.1371/journal.pone.0319109)
Supplement: S3 Data — (DOCX) [file pone.0319109.s003.docx]

**SKILL-SET CHECKLIST**

Name:

Age/Sex:

1. **Team Viewer and V see:**

| **S. No** | **Skill** | **Yes** | **No** |
| --- | --- | --- | --- |
| 1. | Ability to connect to the internet |  |  |
| 2. | Ability to switch on/off the video and audio Ability to connect to the audiologist and speech language pathologist using team viewer app and V see app |  |  |
| 3. | Ability to switch on/off the video and audio |  |  |

1. **Video- otoscopy:**

| **S. No** | **Skill** | **Yes** | **No** |
| --- | --- | --- | --- |
| **1.** | To identify parts of the otoscope   1. Magnifier 2. Speculum 3. Light button |  |  |
| **2.** | Can you prepare for video-otoscopy? |  |  |
| **3.** | Can you place the probe in the ear for otoscopy?   1. For right ear 2. For left ear |  |  |
| **4.** | Show me how you will improve image quality? |  |  |

1. **Pure tone audiometry (PTA):**

| **S. No** | **Skill** | **Yes** | **No** |
| --- | --- | --- | --- |
| **1.** | To identify components of PTA   1. USB cable 2. Headphone 3. Bone vibrator 4. Response button |  |  |
| **2.** | Can you place headphones on this child and show? |  |  |
| **3.** | Can you show me how you will ensure that the headphone fits well? |  |  |
| **4.** | Can you place bone vibrator in left ear? |  |  |
| **5.** | Can you instruct the patient for pure tone audiometry testing? |  |  |

1. **Otoacoustic emission (OAE):**

| **S. No.** | **Skill** | **Yes** | **No** |
| --- | --- | --- | --- |
| **1.** | To identify the parts of OAE   1. USB cable 2. Probe tip 3. Probe wire |  |  |
| **2.** | Connecting to neurosoft equipment to the laptop. |  |  |
| **3.** | Can you select an appropriate probe tip size and insert into the ear. |  |  |

1. **Auditory Brainstem Response (ABR):**

| **S. No** | **Skill** | **Yes** | **No** |
| --- | --- | --- | --- |
| 1. | To identify the parts:   1. Insert wire 2. Insert tips 3. Electrode wires 4. Jumper 5. Button electrodes |  |  |
| 2. | Ability to plug the insert wire appropriately to the neurosoft equipment |  |  |
| 3. | Connection of insert tip to the white connector |  |  |
| 4. | Ability to plug-in the electrode wires and jumper to the neurosoft equipment |  |  |
| 5. | Can you show how will you prepare the child’s skin? |  |  |
| 6. | Can you show how will you connect the electrode wire to the button electrode |  |  |
