# Supplementary material for: Pilot implementation outcomes of a community-based tele- practice model for identification and rehabilitation of children with hearing loss within a public-health system of a Rural District in Southern India
Source: PLoS One. 2025 Mar 19;20(3):e0319109. doi: 10.1371/journal.pone.0319109 (PMC11922231; doi:10.1371/journal.pone.0319109)
Supplement: S4 Data — (DOCX) [file pone.0319109.s004.docx]

**LABEL THE PARTS OF THE EQUIPMENT**

**Video-Otoscopy:**

1. Label the parts of the otoscope.


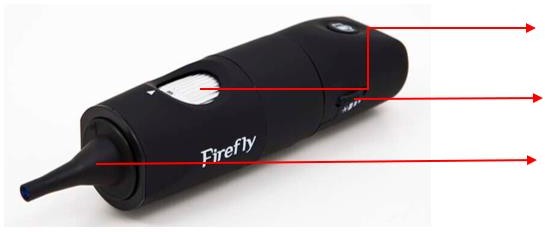
A B C

A -

B -

C -

**Pure-Tone Audiometry**:

A. Identify the following parts:

1. Identify the USB cable from the picture:


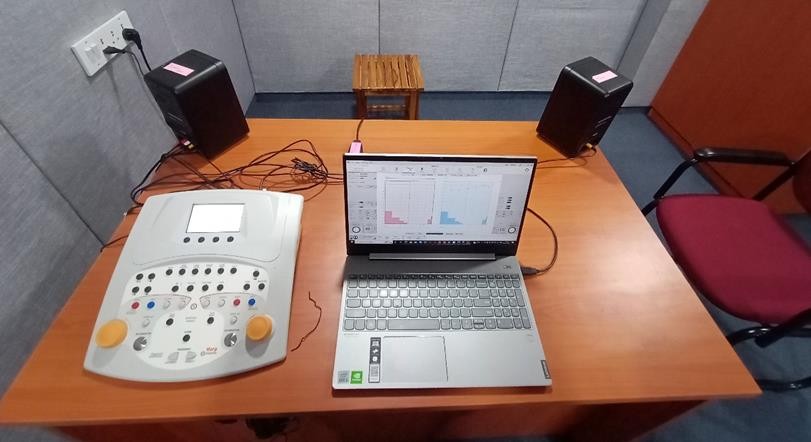


1. **Label the following parts:**

A B C


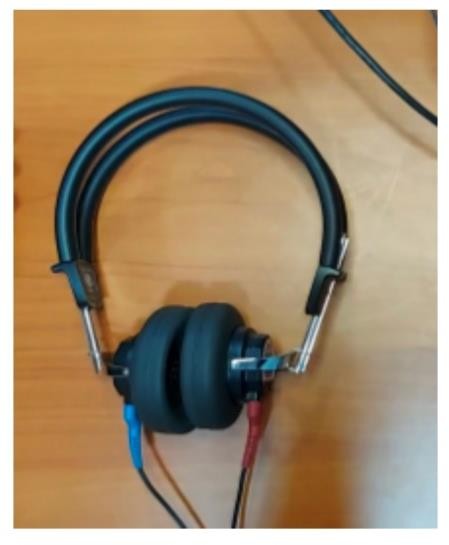

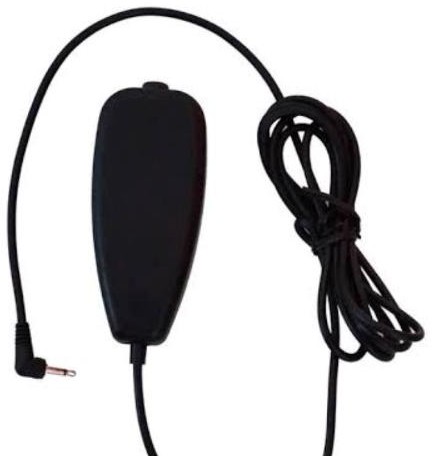

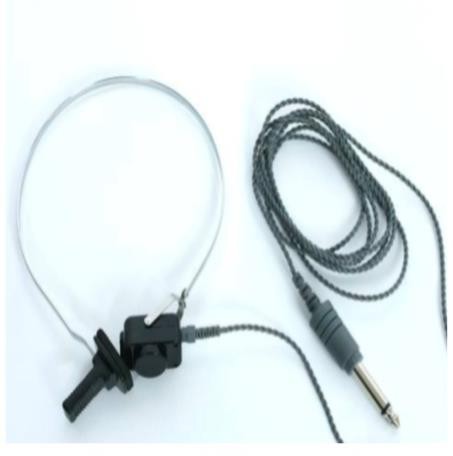


A -

B -

C -

**Otoacoustic emission:**

1. Label the following parts:

A


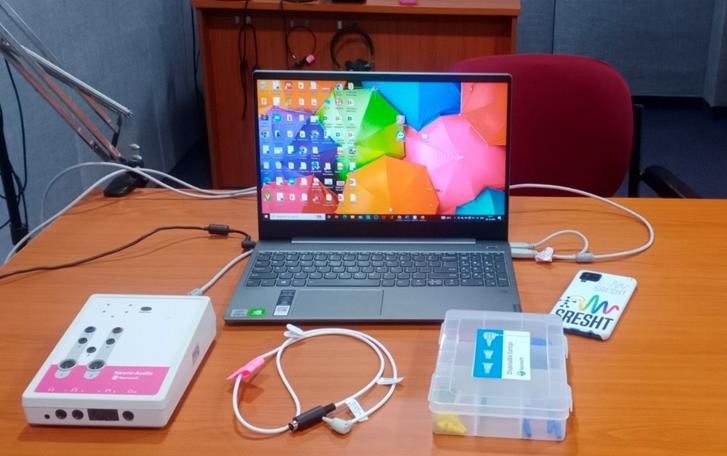


B

C

A -

B -

C -

**Auditory Brainstem Response:**

1. Label the following parts


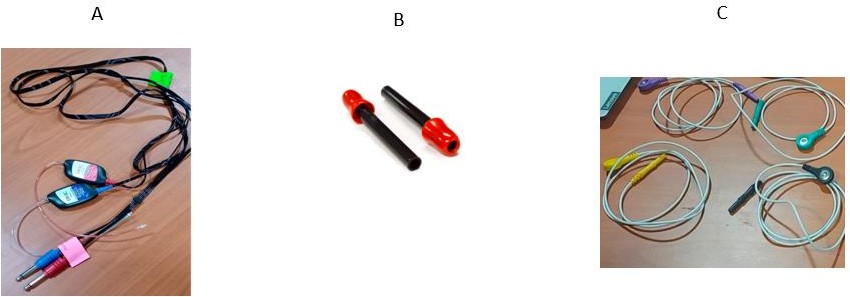


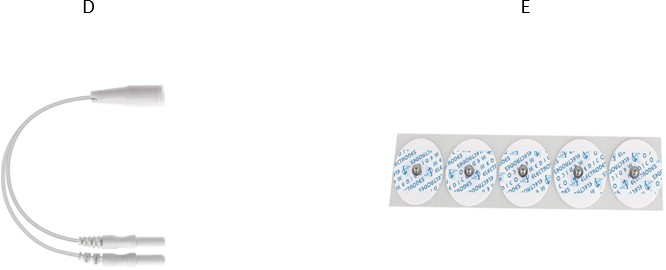


A -

B -

C -

D -
