# Supplementary material for: Pilot implementation outcomes of a community-based tele- practice model for identification and rehabilitation of children with hearing loss within a public-health system of a Rural District in Southern India
Source: PLoS One. 2025 Mar 19;20(3):e0319109. doi: 10.1371/journal.pone.0319109 (PMC11922231; doi:10.1371/journal.pone.0319109)
Supplement: S5 Data — (DOCX) [file pone.0319109.s005.docx]

**TELE-DIAGNOSTIC SATISFACTION QUESTIONNAIRE**

**Name of the child:**

**Age/sex:**

1. **Where was the testing conducted for your child?**

a. Mobile van near the upgraded PHC

b. EIC, Perambalur

c. Room inside upgraded PHC

d. Others (please specify)-

1. **What test was conducted for your child in the mobile-van?**
2. Hearing testing
3. Speech language testing
4. Both a and b

**3**. **Why did you bring your child for testing?**

1. Sent by PHC nurse
2. Sent by GH doctors
3. Sent from Early intervention Centre by Suseela
4. I came on my own
5. Others (please specify)

**4**.**Did you have any hesitation in coming for the testing?**

1. Yes
2. No

 If yes, Give reason-

**5.The audiologist/speech therapist who tested was not in front of you, but tested remotely from elsewhere using a laptop and video-call. How was this experience?**

1. Very poor experience
2. Poor experience
3. Good experience
4. Very good experience

**6. Were you able to see and hear the audiologist/ Speech Therapist satisfactorily through the video-call on the laptop?**

1. Yes
2. No

    If No, Give reason-

**7.Did you need any help from tele-facilitators (Suseela/Aarthi) in asking your questions?**

1. Yes
2. No

**8. Were you able to understand what the audiologist/speech therapist explained to you or advised**?

1. Yes
2. No

**9.Did you have any difficulty in the testing?**

1. Yes
2. No

 If yes, please explain

**10. If this tele-testing facility in mobile-van/ or in EIC/ PHC was not there, where would you have gone for this testing?**

(Specify the place of the testing)

**11. In your opinion, should testing via tele-practice (mobile-van/ at EIC/ at PHC) be continued in your district or would you prefer to go to hospital in nearby cities?**

1. Testing via telepractice to be continued
2. Prefer to go to hospital in nearby cities
3. I don’t want my child to undergo any testing
