# Supplementary material for: Pilot implementation outcomes of a community-based tele- practice model for identification and rehabilitation of children with hearing loss within a public-health system of a Rural District in Southern India
Source: PLoS One. 2025 Mar 19;20(3):e0319109. doi: 10.1371/journal.pone.0319109 (PMC11922231; doi:10.1371/journal.pone.0319109)
Supplement: S6 Data — (DOCX) [file pone.0319109.s006.docx]

**TELE-REHABILITATION SATISFACTION QUESTIONNAIRE**

**Name of the child:**

**Age/sex:**

1. What is your level of satisfaction with the clarity of speech of the therapist through the audio-video call?
   1. Dissatisfied
   2. Satisfied
   3. Very satisfied

2.  What is your level of satisfaction in communicating with the therapist through audio-video call?

1. Dissatisfied
2. Satisfied
3. Very satisfied

3.  Does this therapy via audio-video call meet your expectations for your child?

1. Does not meet my expectations at all
2. Meets my expectation
3. Highly meets my expectation

4. Will you recommend this tele-therapy to others?

1. Yes
2. No

5. What is the level of ease that you perceived in asking questions and expressing your concerns via video-call to the therapist?

1. Difficult
2. Easy
3. Very easy

6. Was the location of the therapy session more suitable than other existing options?

 1- Yes

 2- No

7. What was your level of comfort with this audio-video call based therapy approach?

1. Uncomfortable
2. Comfortable
3. Very comfortable

 8. How likely are you to continue this audio-video call based therapy approach?

1. Unlikely
2. Likely
3. Very likely

9. How well do you recognize the instructions given by the therapist through the laptop?

1. Did not recognize
2. Recognized
3. Very well recognized

10. To what extent did you trust the therapist through Internet communication?

1. I do not trust
2. I trust
3. I very well trust
